# A Systematic Method of Interconnection Optimization for Dense-Array Concentrator Photovoltaic System

**DOI:** 10.1155/2013/275169

**Published:** 2013-12-17

**Authors:** Fei-Lu Siaw, Kok-Keong Chong

**Affiliations:** Faculty of Engineering and Science, Universiti Tunku Abdul Rahman, Off Jalan Genting Kelang Setapak, 53300 Kuala Lumpur, Malaysia

## Abstract

This paper presents a new systematic approach to analyze all possible array configurations in order to determine the most optimal dense-array configuration for concentrator photovoltaic (CPV) systems. The proposed method is fast, simple, reasonably accurate, and very useful as a preliminary study before constructing a dense-array CPV panel. Using measured flux distribution data, each CPV cells' voltage and current values at three critical points which are at short-circuit, open-circuit, and maximum power point are determined. From there, an algorithm groups the cells into basic modules. The next step is *I-V* curve prediction, to find the maximum output power of each array configuration. As a case study, twenty different *I-V* predictions are made for a prototype of nonimaging planar concentrator, and the array configuration that yields the highest output power is determined. The result is then verified by assembling and testing of an actual dense-array on the prototype. It was found that the *I-V* curve closely resembles simulated *I-V* prediction, and measured maximum output power varies by only 1.34%.

## 1. Introduction

Nonuniform flux distribution is a common problem that can be found in all solar concentrator systems [1, 2]. Some of the main contributors to nonuniform illumination are limitation in the design of concentrator optics, slope errors in concentrator profile, tracking error, misalignment of concentrator, and the condition of refractive lens or reflecting mirrors. Some of the causes mentioned such as concentrator optics design and improper tracking could be minimized by implementing new optical design and using improved tracking methods, other causes such as the condition of refractive lens or reflecting mirrors are inevitable defects that are introduced while manufacturing and installation or due to aging. The defects include discoloration of concentrator optics, shape changing, and mechanical fatigue, buckling, and warping [3].

A concentrator photovoltaic (CPV) system performance is affected when there is nonuniform illumination especially for densely packed CPV cells array. When the array is operating under nonuniform illumination, current mismatch will happen among the cells that are connected in series, causing degradation to output power [4]. In single optic/single cell CPV systems that have optical units that are all reasonably well aligned and hence produce the same incident power to all individual CPV cells, current mismatch problem is less critical. In a large array of CPV receivers, usually Fresnel lens system, an additional secondary optical element (SOE) such as flux homogenizer is added to produce uniform illumination [5, 6]. The optical homogenizers that produce uniform flux distribution over solar cells minimize conversion losses caused by chromatic aberration and surface voltage variation. Nevertheless, the additional SOE increases manufacturing cost and the complexity of solar concentrator system [7, 8].

Another method of improving system performance is by adopting nonconventional geometry of CPV cells, in an attempt to improve optical mismatch. For example, a radial large area Si-cell receiver uses custom-shaped cells that divide the incident flux evenly between the cells, as discussed by Vivar et al. [9]. It was presented that the losses from nonuniformity and misalignment decrease by nearly 6 times lesser as compared to a full series connection [10]. However, this method is still vulnerable to tracking errors and optical misalignment. On the other hand, AZUR SPACE Solar Power GmbH also developed custom-sized dense-array modules for the application in parabolic concentrator systems. In their design, dense-array modules consisting of four different geometries of solar cells are arranged in a manner that compensates inhomogeneous illumination. As an example, the outer section of the array that receives lesser light is compensated by using wider segments of CPV module. To avoid higher investment cost from having too many uniquely-sized CPV modules, it is finally reduced from four to two different solar cell types throughout a dense-array [11]. Needless to say, this approach compromised optical matching of the modules. Segev and Kribus introduced High-Voltage silicon Vertical Multijunction (VMJ) cells that were designed for parallel connection in a dense-array [12]. With a parallel connection, voltage matching rather than series matching is attempted to reduce mismatch losses under nonuniform illumination. The new VMJ modules exhibit greater tolerance to nonuniform illumination and tracking errors. Despite the advantages of the new cells, a dense-array that is interconnected in parallel rather than in series will yield a high array current because current from each CPV module is added up. The effect of high array current to resistive losses needs to be further studied to ensure that the overall system efficiency is not jeopardized.

For a CPV system to be cost effective, the whole system should be designed to operate optimally. In fact, a CPV dense-array's interconnection should be arranged according to solar flux distribution pattern of solar concentrator system. In 1963, optical and electrical design considerations were first introduced by Tallent as a basic guideline to predict the performance of a CPV panel for V-trough systems [13]. Nevertheless, this study only covers dense-array CPV panel operating under low concentration and does not discuss nonuniform ity problem. Addressing this need, a systematic method of optimizing performance of dense-array concentrator photovoltaic system under nonuniform illumination is proposed. In addition, this paper also introduces a new fast prediction model of CPV cell using three-point model (TPM) to analyze large and complicated interconnected dense-array cells. The TPM approximation method is fast, and reasonably accurate for optimization purposes, before we go for the comprehensive *I-V* curve simulation. In our method, we can optimize the performance of dense-array CPV system via best electrical interconnection of solar cells for any concentrator such as parabola, lens, and nonimaging concentrator with the use of any standard CPV cells in the market. The procedure of our method is described as follows. First, a dense-array size of any standard sized CPV cells available in the market is estimated based on the flux distribution of a solar concentrator. The array of cells then goes through a computer algorithm that can automatically reconfigure the array of CPV cells in all possibilities and then estimating the output power for each possible configuration. By comparing the output power predicted by the algorithm, a dense-array is selected for assembly. As a case-study, we applied this systematic approach to design and develop an optimized dense-array for a nonimaging planar concentrator. The finalized assembly is installed onto a concentrator prototype, and results such as current-voltage (*I-V*) curve, maximum power (*P*_mp_) and fill factor (FF) are compared to the TPM prediction model.

## 2. Materials and Methods

**Figure 1:**
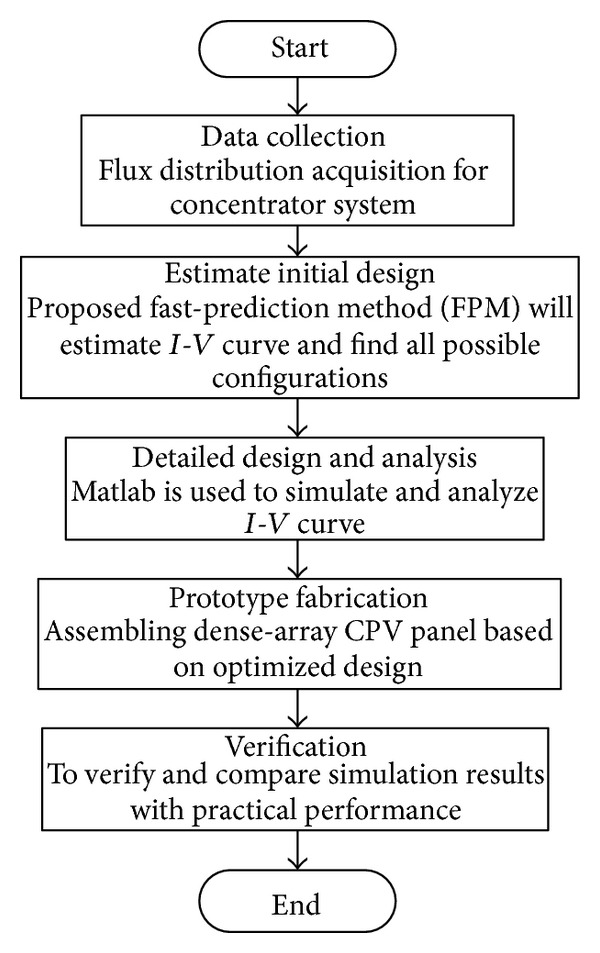
An algorithm to show systematic and complete approach in achieving high-performance dense-array using a newly proposed fast prediction method (FPM) at the second process, which is initial design phase.

**Figure 2:**
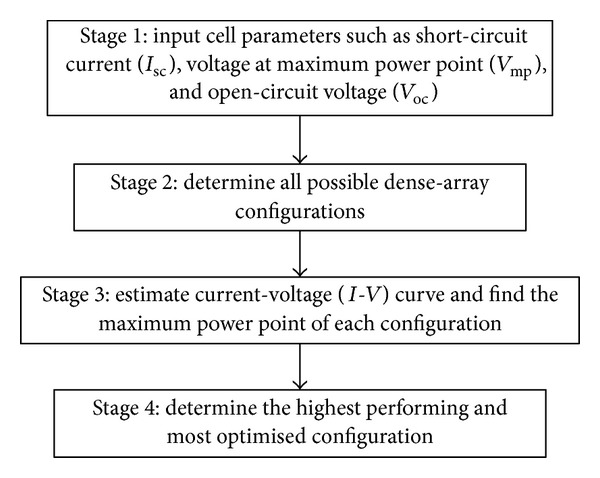
A detailed description showing all four stages of FPM.

Designing CPV dense-array for a concentrator system is a fairly complex process. Conventionally, a trial and error practice that is very dependent on a designer's approach is used to estimate initial design. After first estimation it is necessary to carry out a comprehensive and detailed design to get preliminary results. If the results are not satisfactory, the first design is discarded and another trial design is started from initial design stage again [14]. This process is repeated for a few times based on the system designer's experience to find other possible trial designs. Finally, the trial designs are analyzed to deterimine the best and most optimized one. The optimized design is then verified by experiments. This iterative process is exhaustive and it takes a long time to come up with a good and optimal dense-array design that suits a solar concentrator system. In this paper, a novel fast-prediction method (FPM) that is both systematic and fairly accurate is proposed to replace conventional trial and error practice based on a system designer's experience, intuition and mathematical analysis. An algorithm of the whole process from start until a satisfactory design is presented in flow chart as shown in Figure 1, while four stages of the newly proposed FPM approach for optimizing dense-array design are shown in Figure 2.

### 2.1. Flux Distribution Measurement

**Figure 3:**
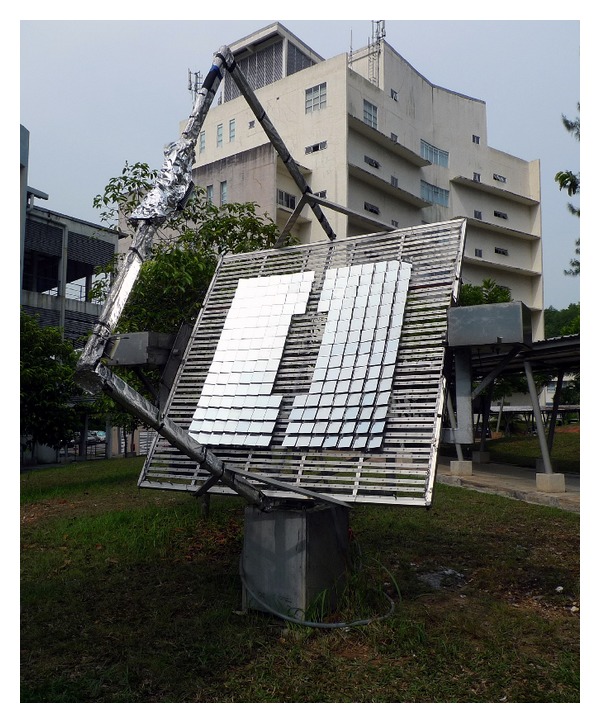
A prototype of nonimaging planar concentrator (NIPC) in the campus of Universiti Tunku Abdul Rahman (UTAR), Malaysia.

Referring to Figure 1, a dense-array design process starts with data collection of the solar concentrator. An NIPC prototype located in Universiti Tunku Abdul Rahman (UTAR) (3.22° North, 101.73° East) is chosen as a case study for this research paper [15]. Using azimuth-elevation sun-tracking method, the concentrator orientation is driven by stepper motors for maintaining its tracking position throughout the day, as presented by Chong and Wong [16]. The concentrator frame holds 192 flat mirrors that are individually prealigned to focus sunlight towards the target. Three outer rings of mirrors as well as some mirrors at the center of the concentrator were not included in this study due to serious blocking between mirrors and shading by the receiver (refer to Figure 3).

**Figure 4:**
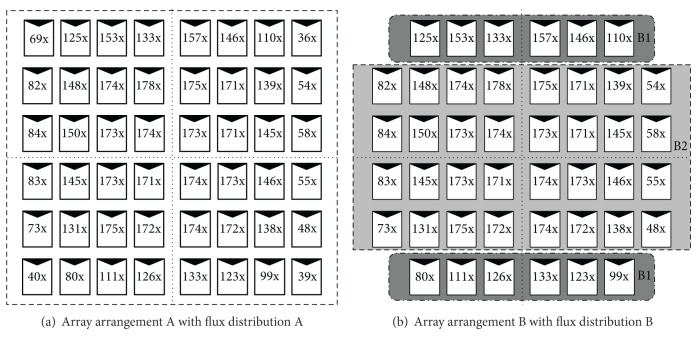
By using measured flux distribution data, the solar concentration ratio at each CPV cell's location is determined. In this study, two dense-array arrangements for two flux distributions are considered, namely, (a) array arrangement A with flux distribution A and (b) array arrangement B with flux distribution B.

After the alignment of optical components is completed, it is essential to measure solar flux distribution on the receiver. An optical scanner equipped with a row of calibrated InGaP/InGaAs/Getriple-junction cells of the size 1.0 cm × 1.0 cm is installed on the receiver to scan along the column direction for retrieving a 2D solar flux distribution. The device setup information of the optical scanner has been presented in our previous publications except the sensors used earlier which were photodiodes with lower limit of irradiance level [17, 18]. During sun-tracking, measurements of flux distribution were made when the image is well focused at the center of the receiver. The measured data are then correlated to absolute irradiance level and presented in Figure 4. Looking at the concentration levels of cells in the Figure 4(a), it is observed that corner cells are exposed to very low solar concentration due to solar disc effect. Since the current of an array follows the lowest current of a series-connected assembly, the corner cells contribute to higher current mismatch, which leads to greater power loss. Hence, current mismatch can be minimized by omitting the corner cells and it might lead to better performance of the overall dense-array. As a comparison between the array with corner cells and array without corner cells, two CPV array arrangements are investigated, namely, (a) array arrangement A with flux distribution A and (b) array arrangement B with flux distribution B.

### 2.2. Development of FPM

**Figure 5:**
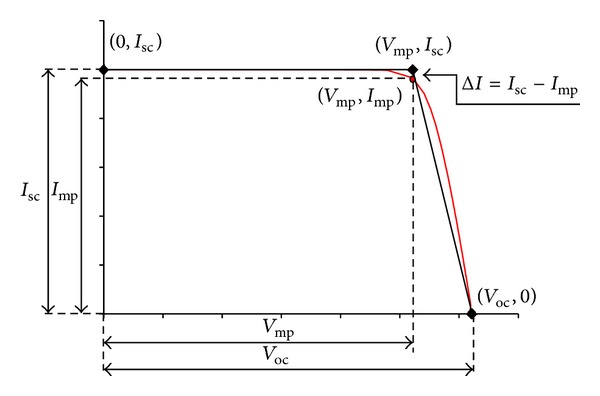
An *I-V* curve of one solar cell (red line) is superimposed with a new TPM prediction model (black line), consisting three critical points, namely, (0, *I*_sc_), (*V*_mp_, *I*_sc_), and (*V*_oc_, 0).

After completing flux distribution data measurements, we proceed to the second process which is to estimate initial design. In this section, we introduce a new approach to formulate the initial design by analyzing TPM *I-V* curve that is useful for the application in a solar concentrator system. The basic principle of the TPM *I-V* curve prediction is to approximate the nonlinear *I-V* curve by using three critical points of each solar cell as presented in Figure 5. In stage 1 of the TPM prediction model, *I-V* curve of each solar cell is represented by three points which are (0, *I*_sc_), (*V*_mp_, *I*_sc_), and (*V*_oc_, 0). The three points consist of the parameters short-circuit current (*I*_sc_), open-circuit voltage (*V*_oc_), and voltage of the maximum power point (*V*_mp_). As observed from the figure mentioned, Δ*I* is very small as the current value at the maximum power point (*I*_mp_) is usually very close (97%-98%) to *I*_sc_. Hence, the TPM model approximates maximum power point to (*V*_mp_, *I*_sc_) instead of (*V*_mp_, *I*_mp_). The rationale of this fast modeling approach is to produce a reasonably accurate approximation model with lesser parameters to save on computing time.

**Figure 6:**
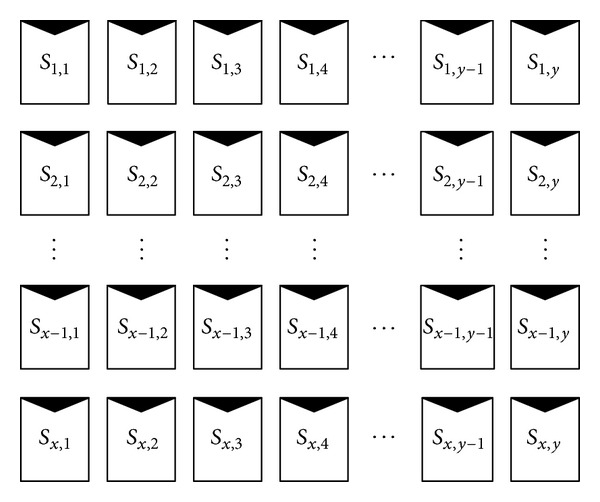
A general network connection of solar cells in an assembly comprising *x* rows and *y* columns of elements.

In a large array consisting of *x* rows and *y* columns of elements, the location of each element/solar cell is represented by *S*_*x*,*y*_ in total-crosstied (TCT) connection (Figure 6). To accurately predict the *I-V* characteristics, solar concentration value (*C*) of each CPV cell is required for retrieving the corresponding parameters such as *I*_sc_, *V*_oc_, and *V*_mp_. These values are then stored in three different matrix files in Matlab environment to be used for sorting.

### 2.3. Determining All Possible Dense-Array Configurations

In our analysis, all solar cells in a basic module are deemed to be connected in parallel and the connection between basic modules is in series. The process of determining all possible dense-array configurations starts by checking the number of cells that are present at the corresponding row. The number of cells in a basic module (*p*) can be calculated as follows:

(1) $$\begin{array}{c} p=\frac{\;N_{\text{cell}}}{d}, \end{array}$$

where *d* is the number of basic modules per row (integer number: 1, 2, 3, etc.) and *N*_cell_ is the total number of cells per row. In this study, we have set that only integer whole numbers of cells are accepted to be used as a basic module. The minimum value of *p* is 1, which means that only one CPV cell forms a basic module and this is the smallest basic module size.

Referring to Figure 4(a), every row in the array consists of eight cells. Using (1), we can calculate every possible number of cells in a basic module for different array configurations. All possible values of *p* for Figure 4(b) are listed in Table 1. From that table, we can see that the cells in region B1 can be connected in four parallel configurations as a basic module, which are six solar cells in parallel (*p*_B1_ = 6), 3 solar cells in parallel (*p*_B1_ = 3), 2 solar cells in parallel (*p*_B1_ = 2), and only one cell in a basic module (*p*_B1_ = 1). On the other hand, the region B2 consists of eight solar cells in parallel (*p*_B2_ = 8), 4 solar cells in parallel (*p*_B2_ = 4), 2 solar cells in parallel (*p*_B2_ = 2), and 1 solar cell in a basic module (*p*_B2_ = 1). As for array arrangement A, the total number of cells in a row is the same throughout the array and thus the values of calculated *p* are similar.

**Figure 7:**
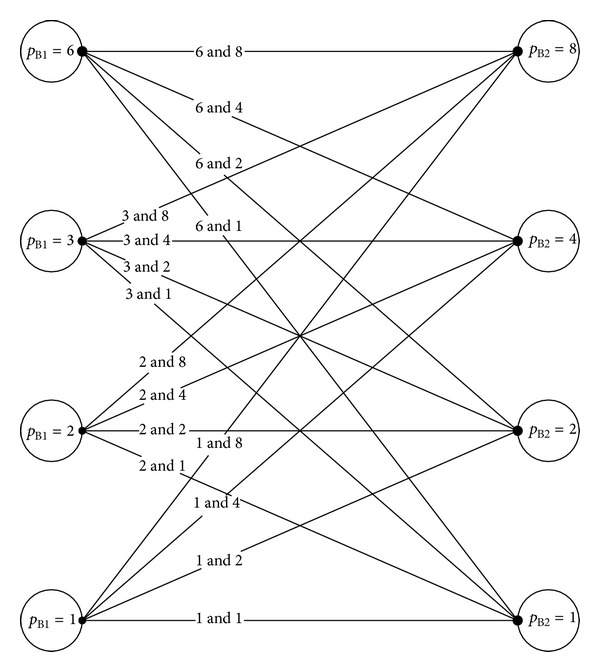
In total, there are sixteen possible configurations for flux distribution B using nodes method.

In flux distribution A, the array consists of equal number of cells in every row. Due to this, series connection is straightforward which are 48 × 1 cells (*p* = 1), 24 × 2 cells (*p* = 2), 12 × 4 cells (*p* = 4), and 6 × 8 cells (*p* = 8). With the configurations mentioned, *I-V* prediction is made. However, flux distribution B (refer to Figure 4(b)) shows that regions B1 and B2 consist of different number of cells in a row. Hence, it is recommended to break the array into two groups which are the arrays that consist of 6 cells in region B1 (top row and bottom row that consists of six cells), and B2 (rows located at the center that consist of 8 cells each). Using the nodes method, a total of sixteen possible configurations are found for array arrangement B (see Figure 7).

### 2.4. Dense-Array Current-Voltage (*I*-*V*) Characteristics Prediction

**Figure 8:**
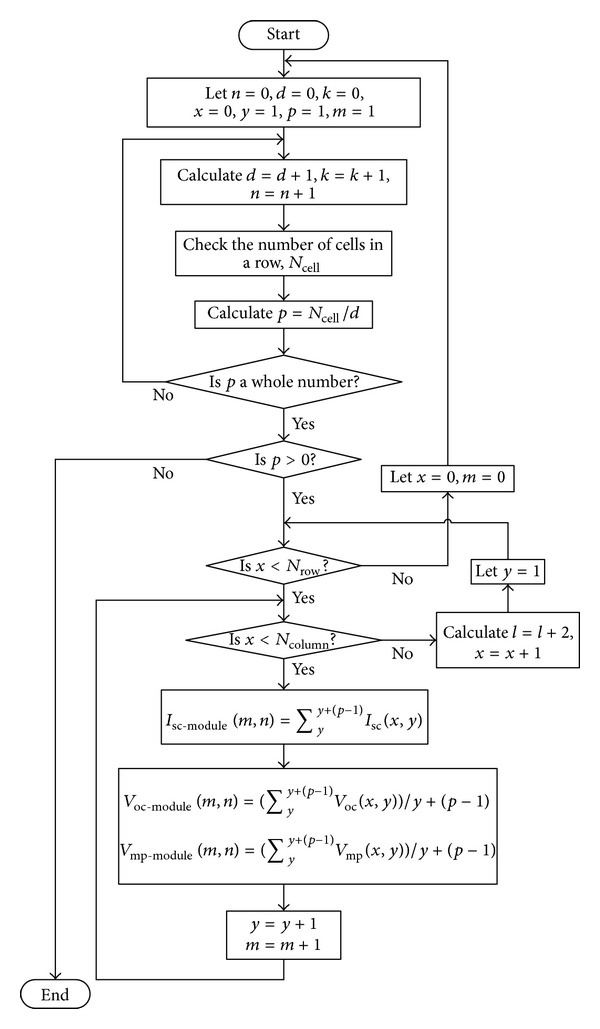
Flowchart of a systematic way of *I*_sc_, *V*_oc_, and *V*_mp_ grouping.

From Figure 8, the flow chart starts by initializing counting parameters that will be used throughout the algorithm. Based on calculated *p*, new values of module short-circuit current (*I*_sc-module_), module open-circuit voltage (*V*_oc-module_), and module voltage at maximum power point (*V*_mp-module_) are calculated row by row until the whole array is completed (see Figure 8). The equations used to calculate the three new parameters can be found in the flow chart, where (*x*, *y*) represents the position of cell at *x*th row and *y*th column of array arrangement as shown in Figure 6, *N*_row_ is the total number of rows, and *N*_column_ is the total number of columns in a CPV array.

Next, module values of the entire solar cell array, that is, *I*_sc-module_ and *V*_oc-module_, are sorted based on decreasing order of *I*_sc-module_ value. The module that produces the highest *I*_sc-module_ is reassigned to *I*_sc-module,*n*_, while its corresponding module open-circuit voltage and module voltage at maximum power point are also reassigned accordingly to *V*_oc-module,*n*_ and *V*_mp-module,*n*_, respectively. Here, *n* is defined as the total number of basic modules in the array configuration. Besides that, this modeling assumes that each basic module is protected with a parallel-connected bypass diode in the opposite polarity. When a basic module receives lower solar irradiance, bypass diode(s) will be forward biased so that the current of the array can safely pass through. When array current passes through bypass diodes, the diodes will turn on and hold its corresponding group of cells to a small negative voltage which will limit any further drop in the total voltage of the array. Bypass diode's forward voltage (*V*_*d*,*n*_) is calculated with the equation

(2) $$\begin{array}{c} V_{d,n}=\left(n-1\right)\times V_{d}, \end{array}$$

where *n* is also the total number of series-connected basic module in an array. *I*_sc-module,1_ is the lowest in the string when *n* = 1 and *V*_*d*,1_ = 0. To complete the *I-V* curve, array open-circuit voltage and array short-circuit current are calculated. At short-circuit condition, array current is equivalent to the highest current value, when voltage is zero (0, *I*_sc-array_); at open-circuit condition, array current is zero and the array voltage is (*V*_oc-array_, 0) as shown in (3). The value of

(3) $$\begin{array}{c} V_{\text{oc-array}}=\sum_{i=1}^{n}V_{\text{oc-module},i}. \end{array}$$

**Figure 9:**
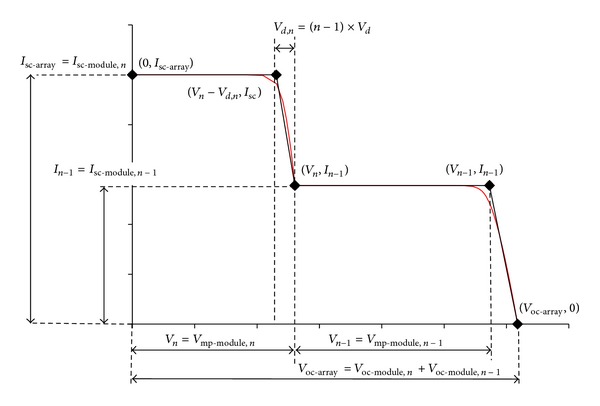
This figure shows critical points of the new approximation model (black line), with two series-connected string in an array (*n* = 2).

**Figure 10:**
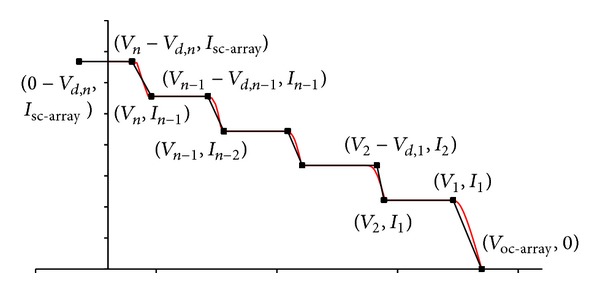
This figure shows the critical points of an array consisting *n* series-connected basic modules.

**Figure 11:**
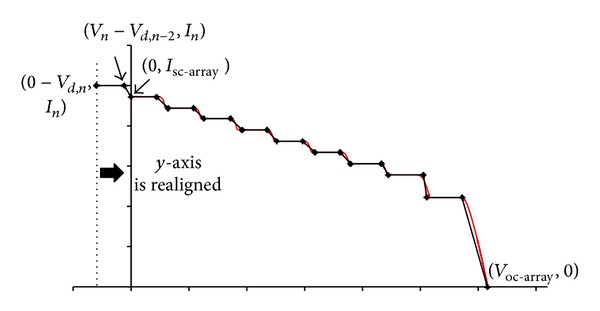
When some critical points lie in the negative voltage region, *y*-axis should be realigned, and *I*_sc-array_ is updated as a current value that crosses the axis.

With all of the values mentioned, critical points of the new array are found. In Figure 9, an example of an array with two modules is presented. In this figure, the output power of each critical point in the array can be calculated by multiplying the voltage to its respective current value. The maximum power (*P*_mp_) of a well-designed array of minimal current mismatch normally occurs on the point (*V*_1_, *I*_1_). For a dense-array with more series-connected modules, an illustration of the *I-V* critical points is presented in Figure 10. For a large series-connected array, some points may appear in the negative voltage, and in these cases *y*-axis is realigned while the value of *I*_sc-array_ is revised to be the array current that crosses the *y*-axis (Figure 11) instead of the highest array current.

The fourth stage of FPM (refer to Figure 2) is to determine the best configuration with the highest performance. In this section, careful analysis is carried out to determine the best option. A summary of simulated *I-V* curve results for both flux distributions based on FPM approach is shown in Table 2. From column *P*_mp_ in the table, it can be observed that the maximum output power from simulation 4 and 6 is the highest among all configurations. Since both output power values are similar, further analysis is necessary. Despite fill factor (FF) being commonly used to evaluate the performance of single solar cell, it does not work the same for dense-array solar cells with *I-V* curve containing multiple current mismatch steps. According to Vorster and Dyk, although FF typically depends on the series and shunt resistance of the cells in the module to relatively reflect the performance quality of the module, the FF does not consider the presence of reverse-bias steps and hence is not useful for measuring the quality of the array *I-V* curve that consists of current mismatched cells [19, 20]. Furthermore, array current cannot be precisely determined using FPM if there is any serious current mismatch in the circuitry.

Power density is another important evaluation criterion when finalizing the initial design of dense-array, and its equation is presented in the following:

(4) $$\begin{array}{c} \text{Power}\;\;\text{density}=\frac{P_{\text{mp}}}{\left(\text{total}\;\;\text{cells}\;\;\text{in}\;\;\text{dense-array}\right)}. \end{array}$$

Referring to the last column of Table 2, power density of simulation 6 is 2.58 W/cell and it is higher than power density of simulation 4 which yields only 2.37 W/cell. This directly indicates that, in average, each solar cell in simulation 6 generates more output power than simulation 4. In fact, the total number of cells in simulation 6 is lesser (44 cells) than that in simulation 4 (48 cells). As simulation 6 is superior in power density while achieving the highest output power among all configurations, it is finally selected.

## 3. Comprehensive Computer Simulations in Matlab

**Figure 12:**
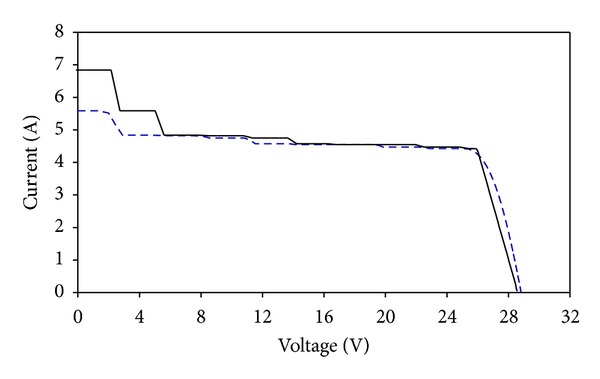
Matlab simulated *I-V* curve (blue dashed line) of the optimized dense-array is superimposed on FPM simulation curve (black line).

**Figure 13:**
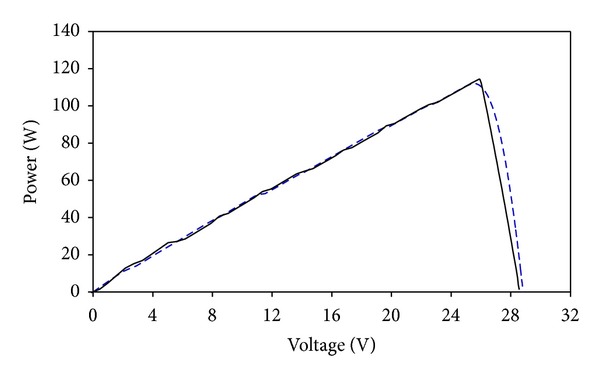
Matlab simulated *P-V* curve of optimized array (blue dashed line) is compared to *P-V* curve of FPM simulation curve (black line).

After the initial design process using FPM approach, a more comprehensive computer simulation is carried out. This detailed simulation includes effects of nonuniform solar distribution and temperature to achieve more accurate *I-V* and *P-V* plots. In our previous publications, a special modeling method using solar cell block from SimElectronics is developed in Matlab to analyze the electrical performance of dense-array [21–23]. For this study, a dense-array with layout configuration of simulation 6 (Table 2) is built in Simulink, and the simulation results are presented in Figures 12 and 13. The simulation was performed for direct normal irradiance (DNI) 641 W/m^2^ and operating temperature 55°C. It was found that the estimation of *P*_mp_ computed from FPM which is 113.64 W is very close to a simulation result from Simulink which is 111.54 W with an error of 1.88%.

## 4. Results and Discussion

**Figure 14:**
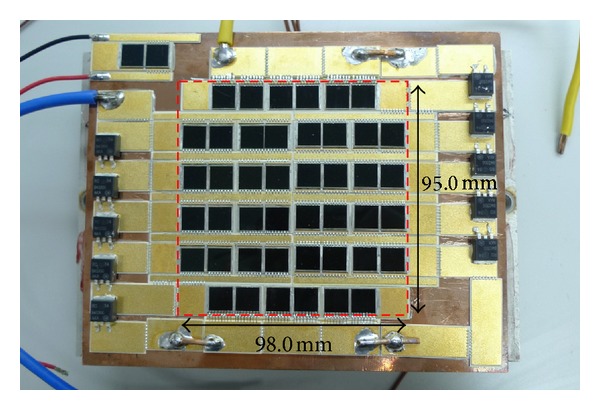
A dense-array CPV assembly of proposed optimized configuration from simulation 6 in Table 2, using triple-junction solar cells [24].

**Figure 15:**
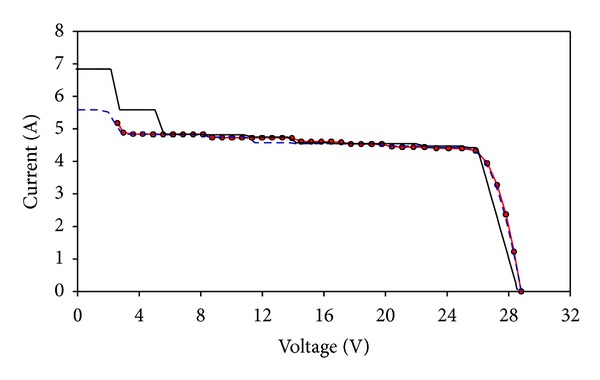
*I-V* curve of measured data (red line with dots) acquired during field test is superimposed on FPM simulation curve (black line) and Matlab simulation curve (blue dashed line).

**Figure 16:**
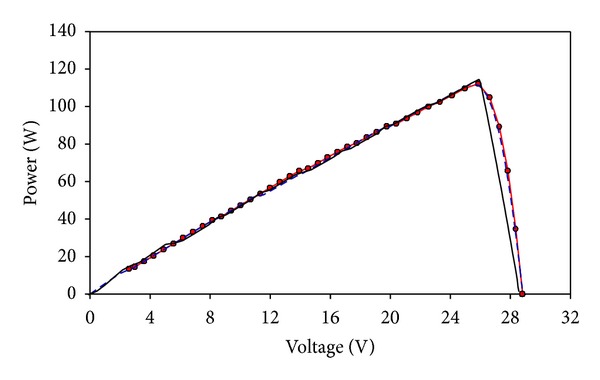
*P-V* curve of the optimized array's measured data (red line with dots) is superimposed on FPM simulation curve (black line) and Matlab simulation curve (blue dashed line).

Using the optimized dense-array configuration, a CPV dense-array is designed and constructed accordingly to confirm our computational modeling results. The dense-array is attached onto a copper cooling block so that operating temperature of the CPV cells can be regulated at about 55°C (refer to Figure 14). Using N3300A configurable DC electronic load mainframe installed with two units of N3305A 500 Watts electronic load modules, real time data acquisition of *I-V* plots was carried out. During *I-V* data acquisition, supporting information such as dense-array operating temperature, direct normal irradiance (DNI), and global irradiance were measured. This study was performed based on real time measured parameters such as DNI and dense-array operating temperature, as well as taking into consideration 15% of optical losses. For comparison purpose, the measured data is superimposed onto simulated *I-V* curve. From Figures 15 and 16, a very close match between measured data and simulated curve is observed, which is only 1.34% of error for *P*_mp_ (refer to Table 3).

Referring to Figures 15 and 16, the *I-V* curve of measured data acquired during field test matches fairly well with FPM prediction curve. The only obvious difference lies around the area from 0 V to 5.6 V. The presence of steps in the prediction curve indicates that current mismatch happened around that voltage region. These steps are not evident in the measured curve as compared to the prediction curve because the combined string current has reduced when some cells are operating at reverse biased condition [25]. As the calculation from the FPM process is a straight-forward addition of current from parallel-connected cells, a clear indication of current mismatch can be seen and this is very helpful for system designers to evaluate the severity of mismatch in a dense-array design. Furthermore current mismatch at the 0 V to 5.6 V range has negligible effect to the *P-V* curve (see Figure 16). Hence, it will not affect the calculation of *P*_mp_ of a well-designed dense-array panel, which normally occurs near to *V*_oc_ region of a *P-V* curve.

## 5. Conclusion

Conventionally, CPV dense-array design is an exhaustive iterative process to achieve a preset output power requirement. This design approach is not comprehensive because designers do not explore all dense-array configuration possibilities. In this study a systematic and complete method is introduced in achieving the most optimal dense-array design using the newly proposed FPM at the initial design phase. The FPM consists of four stages and is developed to optimize dense-array configurations through a systematic approach instead of conventional trial and error method. The first stage is where cell parameters such as *I*_sc_, *V*_mp_, and *V*_oc_ are calculated from measured flux distribution data. After that, every possibility of array configurations is predicted at the second stage of FPM. The third stage deals with *I-V* curve prediction using critical points of solar cells and bypass diodes that are connected across each basic module. Finally, the *I-V* prediction curve is analyzed by comparing with the calculated *P*_mp_. This four-stage approach is very systematic, fast and capable to explore all possibilities of dense-array configurations, while maintaining reasonable accuracy. From this method, an optimized configuration in simulation 6 (Table 2) was found to have the highest output power, together with simulation 4. Upon further evaluation, simulation 6 was selected because its power density is superior (2.58 W/cell) as compared to the calculated power density in simulation 4 (2.37 W/cell). By optimizing dense-array layout configuration, simulation 6 with only 44 cells can achieve the same output power as simulation 4 with 48 cells. When lesser solar cells are used, a system designer is able to reduce installation cost while increasing the competitiveness of concentrator solar technology. This finding highlights a new important factor that affects power density which is layout configuration, in addition to the influence of solar concentration. At the same time, it was found that FF is not a conclusive benchmark when evaluating dense-array solar cells. While FF is commonly used to evaluate the quality and performance of a single solar cell, it can only act as a guideline and not a deciding factor when finalizing dense-array design. This can be confirmed when we compare the results of simulation 6 and simulation 4 listed in Table 2. While the FF of simulation 4 is higher (68.95%) as compared to simulation 6 (57.71%), simulation 4 requires more CPV cells to generate the same amount of power as simulation 6. Once the initial design of dense-array has been completed, detailed computer simulations are carried out to verify the prediction. Comprehensive simulation using Matlab has verified the proposed FPM prediction as presented in Section 3 with the value of *P*_mp_ having only 1.88% in error. Last but not least, an actual assembly of the dense-array was built and installed on an NIPC prototype. The modeling method had been successfully validated with the NIPC prototype to achieve practical conversion efficiency of 34.19% with similar *I-V* curve characteristics. Comparing the results obtained from field measurement with FPM simulated results by evaluating *I-V* and *P-V* curves, a very close match can be observed. It was found that the estimation of *P*_mp_ by computational modeling is only 1.34% in error.

## Figures and Tables

**Table 1:** Calculation of *p* for flux distribution *B*.

Integer (*d*)	*p*_B1_ = *N*_cell_/*d* (In region B1, *N*_cell_ = 6)	*p*_B2_ = *N*_cell_/*d* (In region B2, *N*_cell_ = 8)
1	6	8
2	3	4
3	2	—
4	—	2
5	—	—
6	1	1
7	—	—
8	—	1

**Table 2:** Comparison of different array configurations at DNI: 641 W/m^2^.

Simulation no.	Array configuration	Flux distribution	*P*_mp_ (W)	Fill factor (FF) (%)	*V*_mp_ (V)	*I*_mp_ (A)	*V*_oc_ (V)	*I*_sc_ (A)	Power density *P*_mp_/no. cells (W/cell)
1	48 × 1	*A *	79.36	39.74	77.77	1.02	137.71	1.45	1.65
2	24 × 2	*A *	82.09	41.82	52.98	1.55	68.88	2.85	1.71
3	12 × 4	*A *	91.24	54.96	30.72	2.97	34.44	4.82	1.90
4	6 × 8	*A *	113.62	68.95	15.36	7.4	17.22	9.57	2.37
5	2 × 6 (B1) and 4 × 8 (B2)	*B *	86.01	53.15	15.4	5.58	17.27	9.37	1.95
6	2 × 6 (B1) and 8 × 4 (B2)	*B *	113.64	57.71	25.68	4.43	28.79	6.84	2.58
7	2 × 6 (B1) and 16 × 2 (B2)	*B *	71.63	20.2	46.22	1.55	51.83	6.84	1.63
8	2 × 6 (B1) and 32 × 1 (B2)	*B *	71.09	10.62	61.86	1.42	97.9	6.84	1.62
9	4 × 3 (B1) and 4 × 8 (B2)	*B *	81.69	38.14	9.07	9	23.03	9.3	1.86
10	4 × 3 (B1) and 8 × 4 (B2)	*B *	85.63	51.33	19.35	4.43	34.54	4.83	1.95
11	4 × 3 (B1) and 16 × 2 (B2)	*B *	79.58	40.29	51.36	1.55	57.58	3.43	1.81
12	4 × 3 (B1) and 32 × 1 (B2)	*B *	76.99	21.66	66.99	1.15	103.65	3.43	1.75
13	6 × 2 (B1) and 4 × 8 (B2)	*B *	76.29	28.29	8.47	9	28.78	9.37	1.73
14	6 × 2 (B1) and 8 × 4 (B2)	*B *	82.97	42.64	18.75	4.43	40.29	4.83	1.89
15	6 × 2 (B1) and 16 × 2 (B2)	*B *	87.53	47.17	56.49	1.55	63.33	2.93	1.99
16	6 × 2 (B1) and 32 × 1 (B2)	*B *	82.88	31.44	72.12	1.15	109.4	2.41	1.88
17	12 × 1 (B1) and 4 × 8 (B2)	*B *	60.09	13.93	6.67	9	46.03	9.37	1.37
18	12 × 1 (B1) and 8 × 4 (B2)	*B *	75.01	26.99	16.95	4.43	57.55	4.83	1.70
19	12 × 1 (B1) and 16 × 2 (B2)	*B *	61.7	26.13	60.47	1.02	80.58	2.93	1.40
20	12 × 1 (B1) and 32 × 1 (B2)	*B *	80.58	42.99	78.97	1.02	126.66	1.48	1.83

**Table 3:** Comparison between simulated (Matlab) and measured results of the dense-array CPV assembly is presented in terms of maximum output power *P*_mp_, maximum voltage *V*_mp_, maximum current *I*_mp_, array efficiency, and error of maximum output power *P*_mp_.

	DNI: 641 W/m^2^		
	*P*_mp_ (W)	*V*_mp_ (V)	*I*_mp_ (A)
Measured results	112.14	25.87	4.33
FPM simulated results	113.64	25.68	4.43

Efficiency measured (%)	34.19		
Efficiency simulated (%)	34.64		
Error, *P*_mp_ (%)	−1.34		
